# Supplementary material for: Viral transduction of primary human lymphoma B cells reveals mechanisms of NOTCH-mediated immune escape
Source: Nat Commun. 2022 Oct 20;13:6220. doi: 10.1038/s41467-022-33739-2 (PMC9585083; doi:10.1038/s41467-022-33739-2)
Supplement: Supplementary file 1 — Supplementary Information [file 41467_2022_33739_MOESM1_ESM.pdf]

Supplementary Figure 1

a

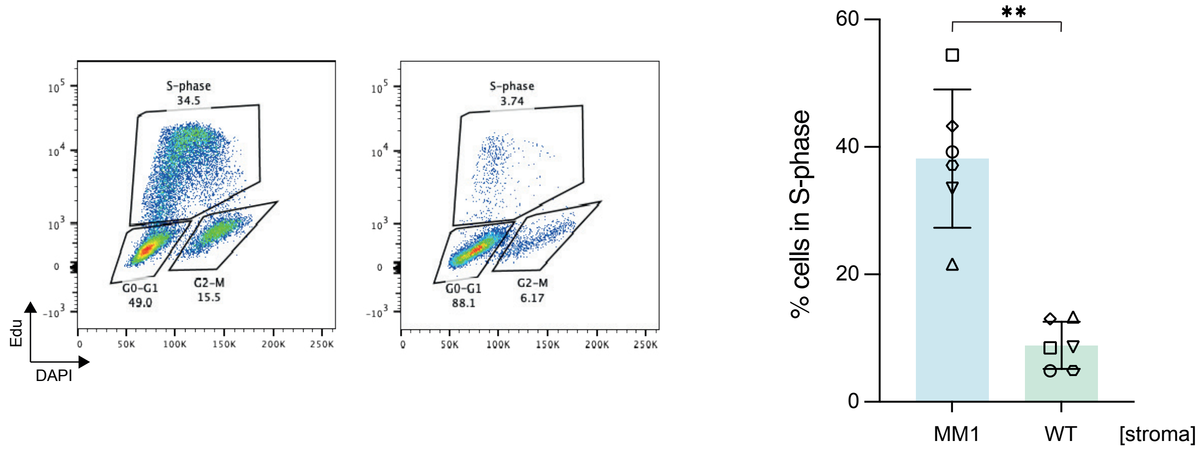

b

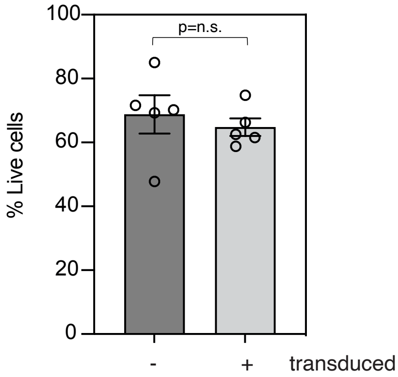

a. Cell cylce analysis of primary CLL cells 5 days after transduction. Cells were either continiously cultured on MM1 cells or re-cultured on wild-type (WT) stroma cells for the last 48 hours of the experiment [n=6]. Error bars are shown as mean± SEM ($p=0.0022$; two tailed paired t-test).

b. Comparison of viable cells between non-transduced and transduced CLL cells cultured under identical conditions. Apoptosis was measured by Annexin V/DAPI staining 3 days post infection [6 days post thawing]. Shown is the mean ± SEM of n=5 with individual primary CLL cells. ns=non significant; paired two-tailed t-test. Error bars are shown as mean ± SEM.

Supplementary Figure 2

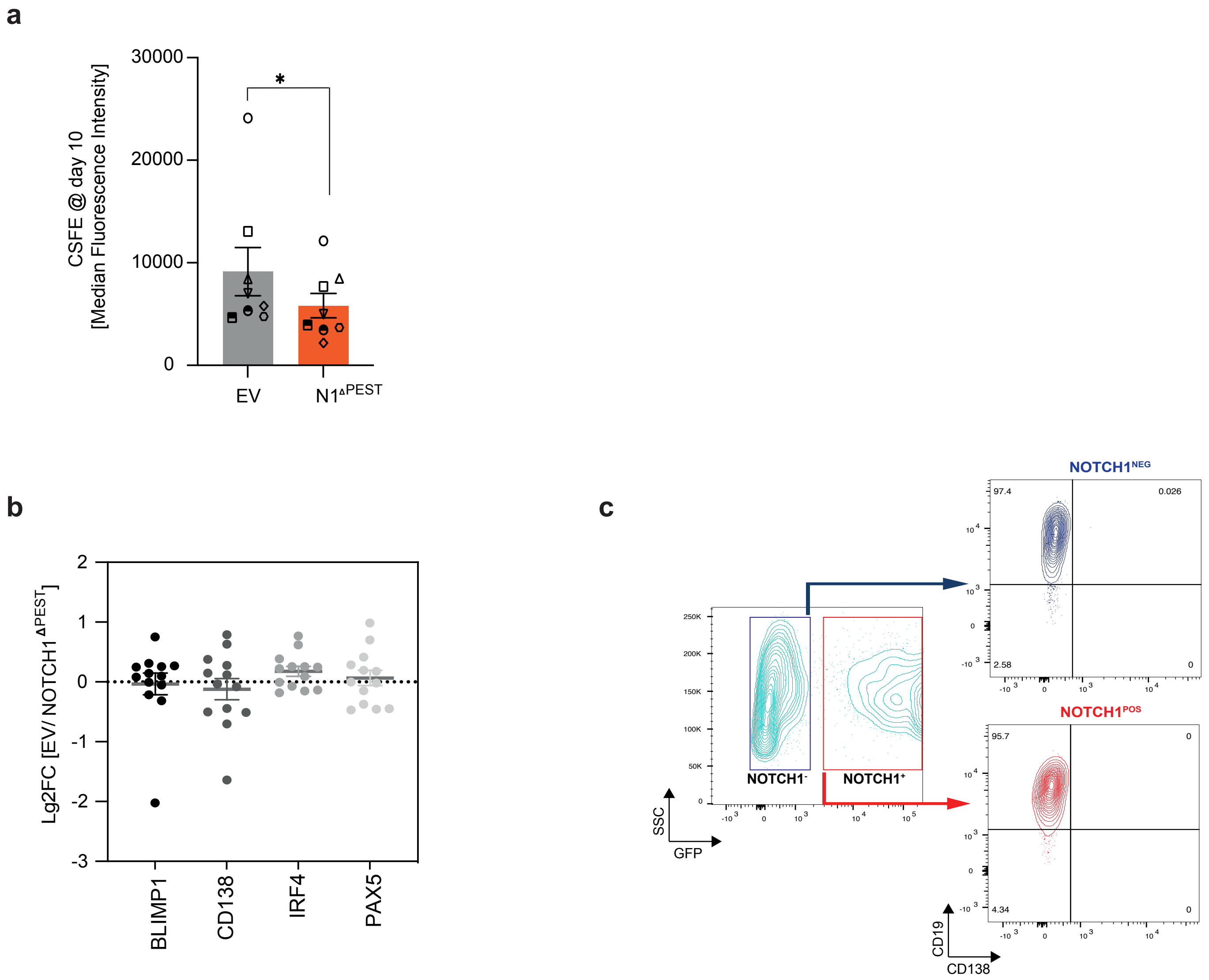

a. Quantification of cell divisions of primary CLL cells 10 days after transduction labelled with the CellTrace™ CFSE reagent. Each symbol represents an individual patient sample (n=8). Error bars are shown as mean ± SEM. Statistical significance was assessed by a paired t-test (p=0.046)

b. Log2FC values of genes associated with plasma cell differentiation, analyzed by RNAseq following NOTCH1$^{\Delta PEST}$ transduction. (n=13 individual patient samples). Error bars are shown as mean ± SEM.

c. CD138 expression on NOTCH1$^{\Delta PEST}$ transduced (red) compared to untransduced CLL cells (blue) 5 days post transduction. Representative flow-cytometry data from a total of 3 individual repeats with different primary cells.

Supplementary Figure 3

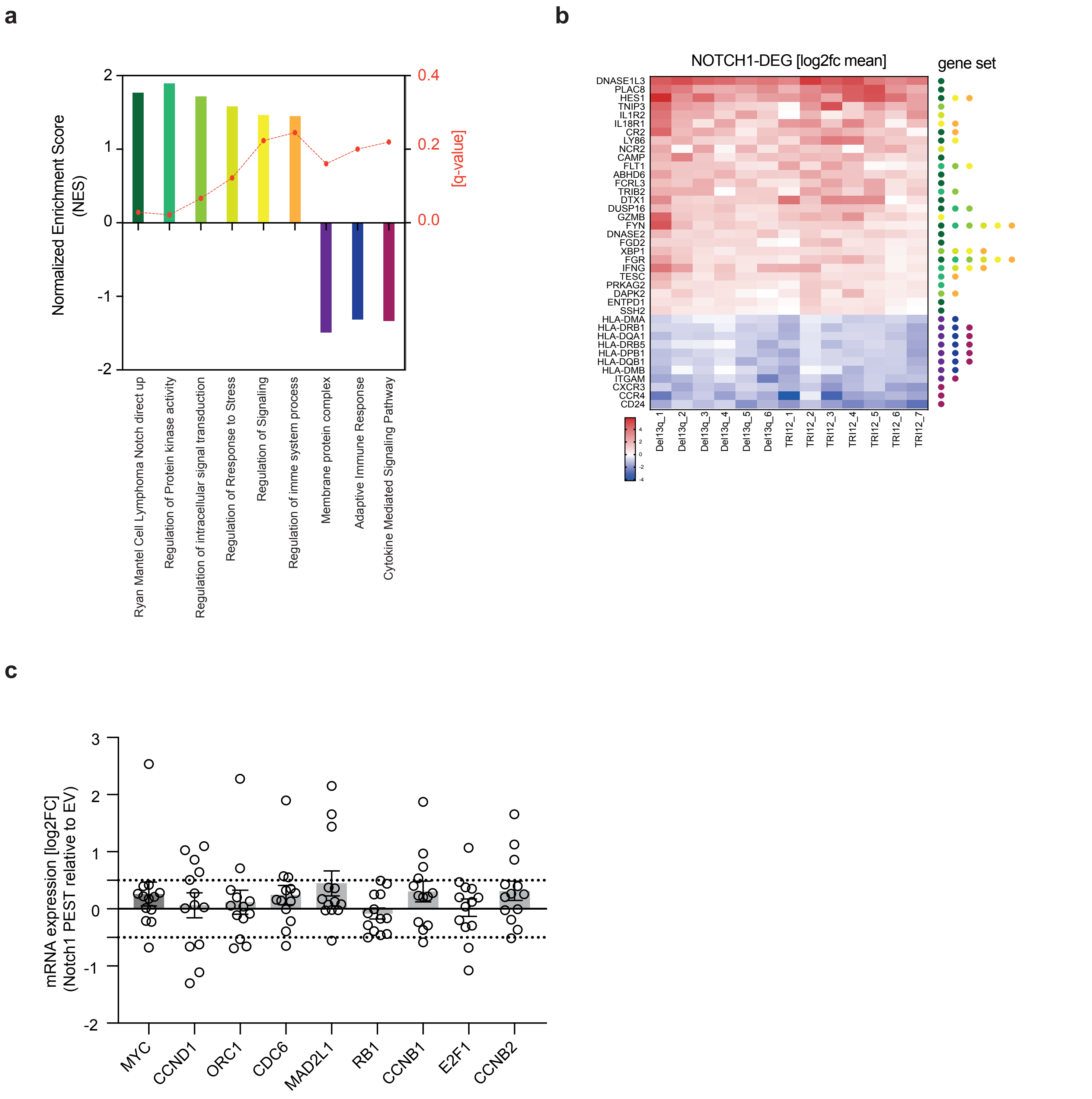

a. Gene set enrichment analysis (GSEA) results of NOTCH1$^{\Delta PEST}$ genes commonly DE in Trisomy 12 and Del13q primary CLL cells.

b. Heatmap of the core genes of the datasets identified in panel a. The identified dataset to which each gene belongs to is shown using a color-coded system, indicated on the right.

c. Bar graph of the Log2FC values of cell cycle related gene expression analyzed by RNAseq following NOTCH1$^{\Delta PEST}$ transduction (n=13). Error bars are shown as mean ± SEM.

Supplementary Figure 4

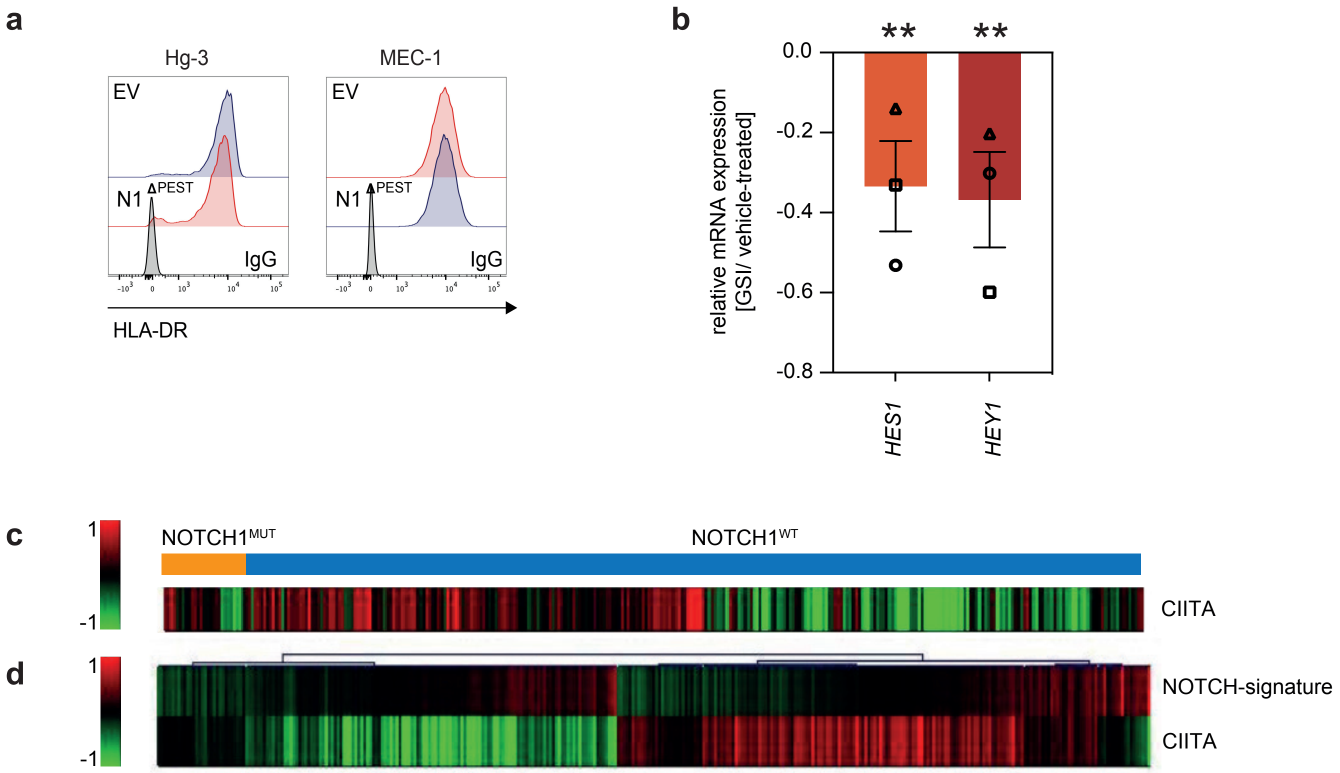

a. Flow cytometry analysis of HLA-DR expression on CLL cell lines (Mec-1 and Hg-3) following expression of NOTCH1$^{\Delta PEST}$. One representative experiment out of three is shown.

b. qRT-PCR analysis of *HES1* and *HEY1* expression in primary *NOTCH1* mutated CLL cells treated with a γ-secretase inhibitor. Expression is normalized to vehicle treated cells. Each dot represents an individual patient sample (n=3). Error bars are shown as mean ± SEM. Statistical significance was determined by a paired t-test (p=0.0071 and p=0.0075)

c. Heatmap showing *CIITA* expression in treatment-naïve NOTCH1-mutated and wild-type CLL patients (n=337).

d. Heatmap of mRNA expression profiles of the averaged expression levels of *HES1/2*-,*HEY1/2*- and corresponding *CIITA* levels in treatment naïve CLL (n=337).

Supplementary Figure 5

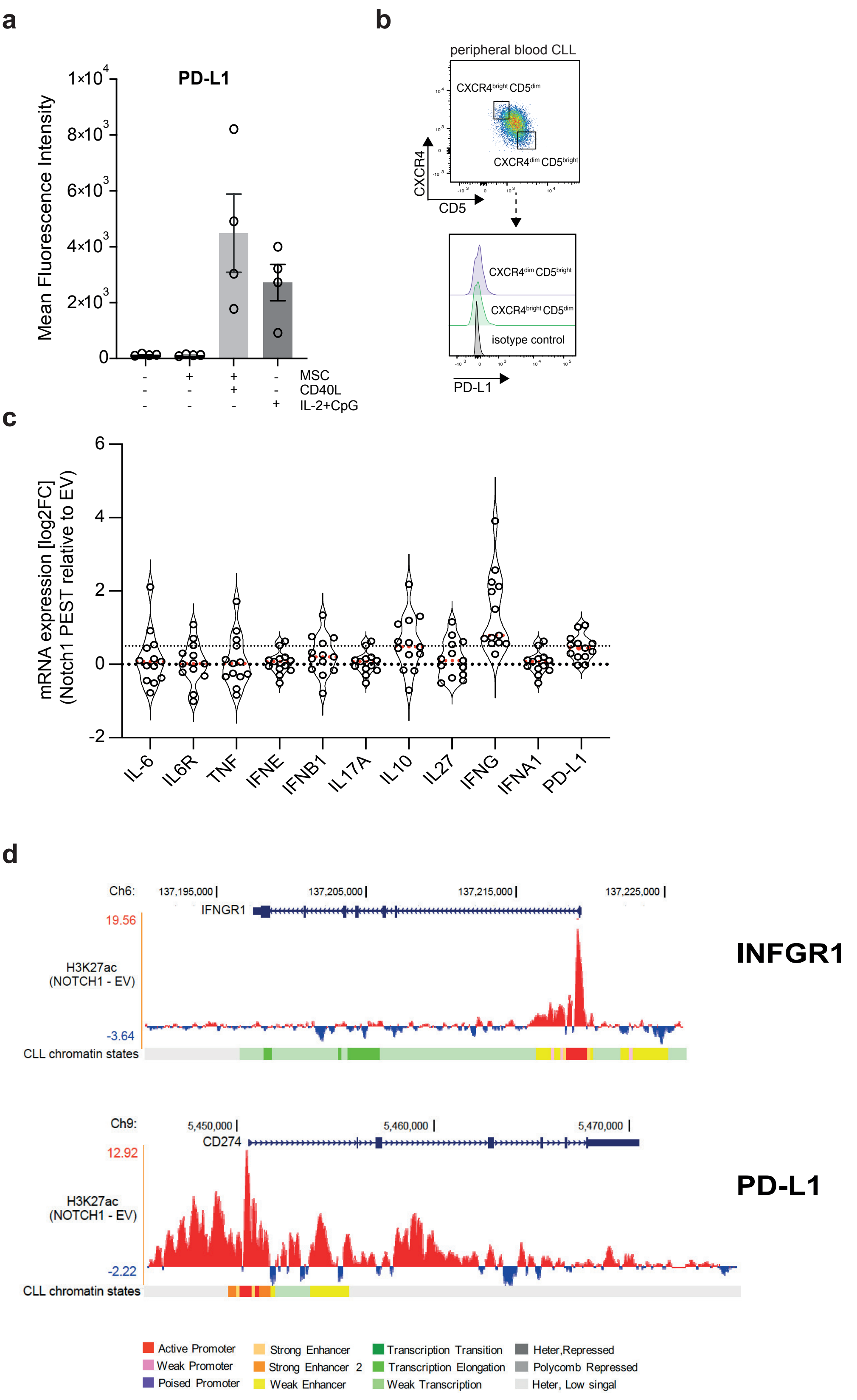

a. Quantification of PD-L1 expression on activated CLL cells cultured in suspension or on CD40L-expressing mesenchymal stroma cells (MSCs) for 48h. Alternatively, cells were stimulated with CpGs (ODN-DSP30 1μM) and IL-2 (100 U/ml) (n=4) to induce proliferation.

b. PD-L1 analysis on freshly isolated, peripheral blood derived CLL cells in $CD19^{+}CXCR4^{dim}/CD5^{bright}$ and $CD19^{+}CXCR4^{bright}/CD5^{dim}$ populations (n=6). Representative data are shown for one patient and illustrate the gating-strategy and expression of PD-L1.

c. Bar graph of the Log2FC values of inflammatory cytokine expression analyzed by RNAseq following $NOTCH1^{\Delta PEST}$ transduction (n=13).

d. H3K27ac Chip-seq profile of *INFGR1* and *PD-L1* following $NOTCH1^{\Delta PEST}$ overexpression. The peaks represent the mean of the ratio of values obtained from CLL cells transduced with $NOTCH1^{\Delta PEST}$ or with an empty vector control (n=5). Each gene and loci locations are shown.

Supplementary Figure 6

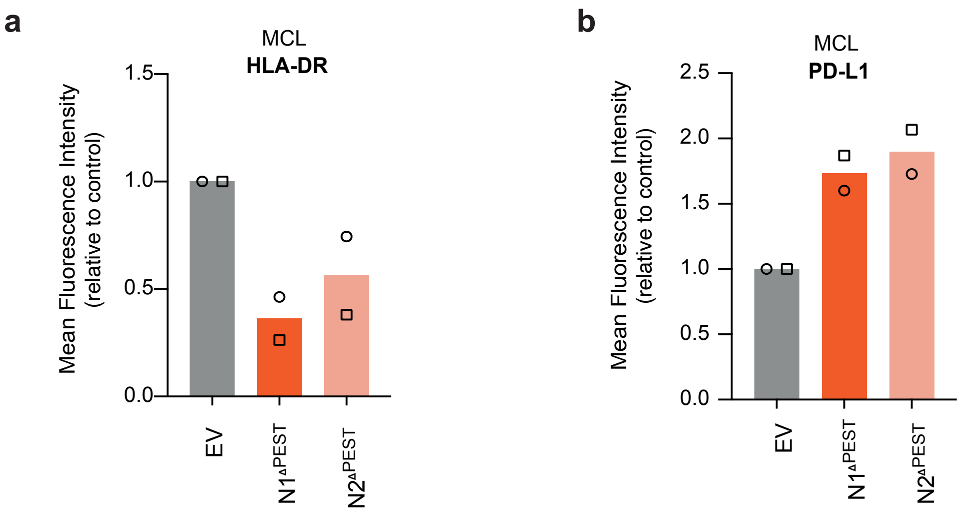

a. HLA-DR expression assessed by flow cytometry on primary MCL cells from n=2 patients, transduced with either truncated NOTCH1$^{\Delta PEST}$ or NOTCH2$^{\Delta PEST}$.

b. PD-L1 expression assessed by flow cytometry on primary MCL cells from n=2 patients, transduced with either truncated NOTCH1$^{\Delta PEST}$ or NOTCH2$^{\Delta PEST}$.

Supplementary Figure 7

a

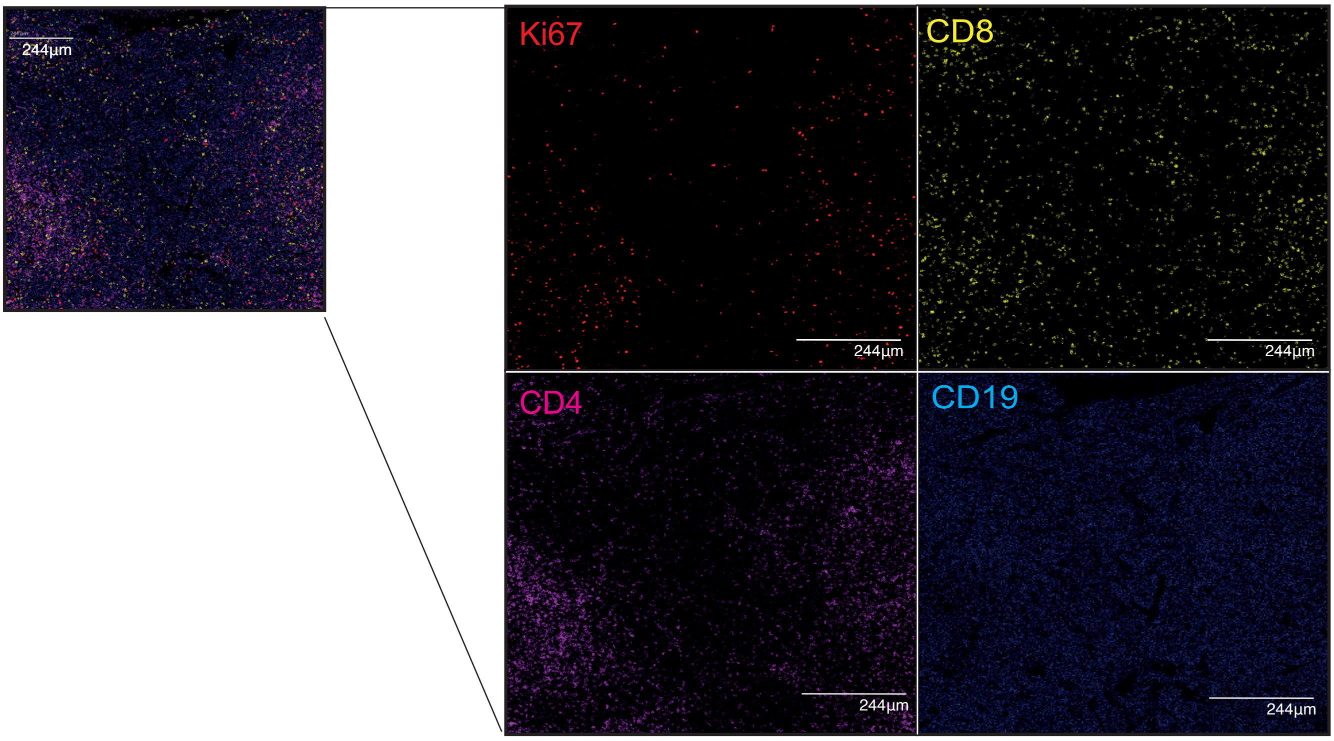

a. Multiplexed IMC image example of a NOTCH1 positive specimen with single channel images (Ki67=Red, CD8=Yellow, CD4=Magenta, CD19=Blue). One representative staining out of a total of 4 analysed individual patient samples is shown.

Supplementary Figure 8

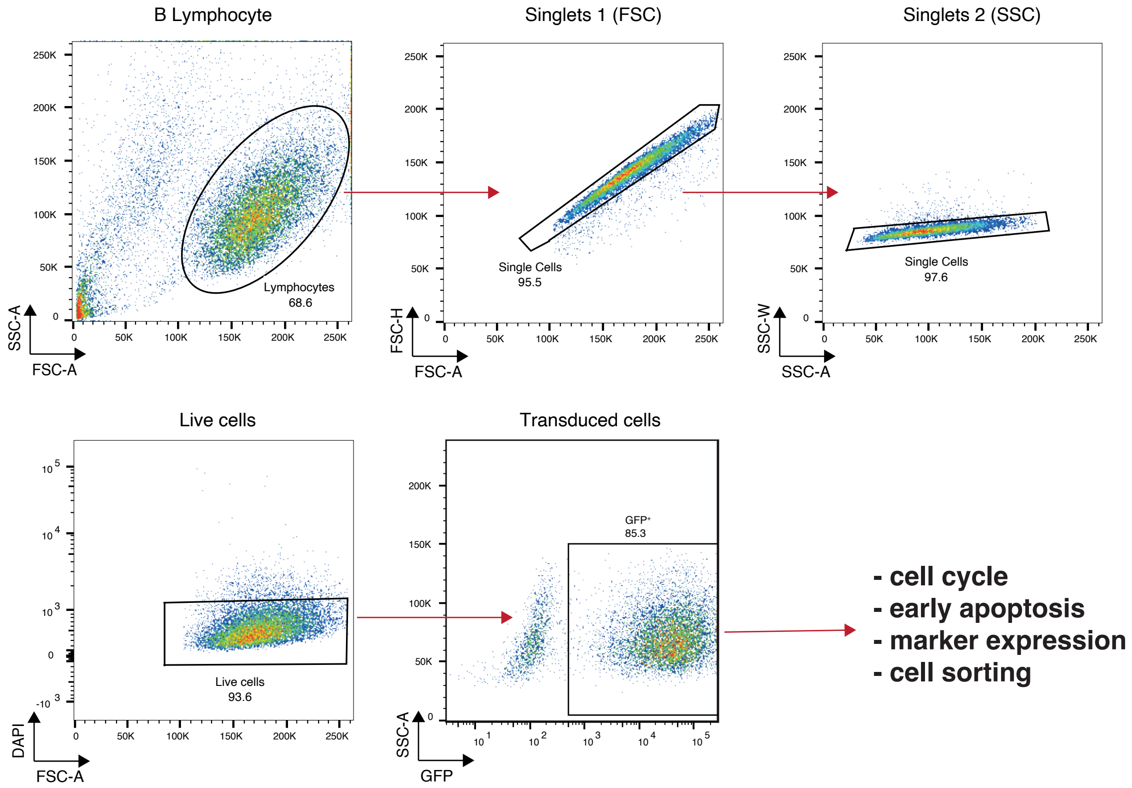

General gating strategy applied in this study. Downstream gating strategy and analysis are shown in each figure of the manuscript for every experiment performed.

Supplementary Table 1: List of primers for qRT-PCR

TARGET GENE	PRIMER 5'-> 3'
HEY1 - Forward	GTTCGGCTCTAGGTTCCATGT
HEY1 - Reverse	CGTCGGCGCTTCTCAATTATTC
HES1 - Forward	ACGTGCGAGGGCGTTAATAC
HES1 - Reverse	CGTCGGCGCTTCTCAATTATTC
DTX1 - Forward	GACGGCCTACGATATGGACAT
DTX1 - Reverse	CCTAGCGATGAGAGGTCGAG
CDKN1A - Forward	ACATCGCCAAGGAAAAACGC
CDKN1A - Reverse	GTCTGTTTCGGTACTGTCATCC
GAPDH - Forward	CCTGTTCGACAGTCAGCCG
GAPDH - Reverse	CGACCAAATCCGTTGACTCC
