# Supplementary material for: Viral transduction of primary human lymphoma B cells reveals mechanisms of NOTCH-mediated immune escape
Source: Nat Commun. 2022 Oct 20;13:6220. doi: 10.1038/s41467-022-33739-2 (PMC9585083; doi:10.1038/s41467-022-33739-2)
Supplement: Supplementary file 3 — Description of Additional Supplementary Files [file 41467_2022_33739_MOESM3_ESM.pdf]

Supplementary Data 1: List of NOTCH1 DE genes (refers to Figure 3d)
Supplementary Data 2: List of NOTCH1-DE H3K27ac peaks (refers to Figure 3f)
Supplementary Data 3: List of NOTCH1 upregulated proteins (refers to Figure 5a)
