# Supplementary material for: Viral transduction of primary human lymphoma B cells reveals mechanisms of NOTCH-mediated immune escape
Source: Nat Commun. 2022 Oct 20;13:6220. doi: 10.1038/s41467-022-33739-2 (PMC9585083; doi:10.1038/s41467-022-33739-2)
Supplement: Supplementary file 7 — Reporting Summary [file 41467_2022_33739_MOESM7_ESM.pdf]

nature research

Corresponding author(s): Dr Ingo Ringshausen

Last updated by author(s): Aug 25, 2022

Reporting Summary

Nature Research wishes to improve the reproducibility of the work that we publish. This form provides structure for consistency and transparency in reporting. For further information on Nature Research policies, see our Editorial Policies and the Editorial Policy Checklist.

Statistics

For all statistical analyses, confirm that the following items are present in the figure legend, table legend, main text, or Methods section.

n/a	Confirmed	
☐	☒	The exact sample size (*n*) for each experimental group/condition, given as a discrete number and unit of measurement
☐	☒	A statement on whether measurements were taken from distinct samples or whether the same sample was measured repeatedly
☐	☒	The statistical test(s) used AND whether they are one- or two-sided<br>*Only common tests should be described solely by name; describe more complex techniques in the Methods section.*
☒	☐	A description of all covariates tested
☐	☒	A description of any assumptions or corrections, such as tests of normality and adjustment for multiple comparisons
☐	☒	A full description of the statistical parameters including central tendency (e.g. means) or other basic estimates (e.g. regression coefficient) AND variation (e.g. standard deviation) or associated estimates of uncertainty (e.g. confidence intervals)
☐	☒	For null hypothesis testing, the test statistic (e.g. *F*, *t*, *r*) with confidence intervals, effect sizes, degrees of freedom and *P* value noted<br>*Give P values as exact values whenever suitable.*
☒	☐	For Bayesian analysis, information on the choice of priors and Markov chain Monte Carlo settings
☒	☐	For hierarchical and complex designs, identification of the appropriate level for tests and full reporting of outcomes
☐	☒	Estimates of effect sizes (e.g. Cohen's *d*, Pearson's *r*), indicating how they were calculated

Our web collection on statistics for biologists contains articles on many of the points above.

Software and code

Policy information about availability of computer code

Data collection	BD Fortessa X-20, BD Influx Cell Sorter, Hyperion Imaging System, Azure Biosystem, Fusion Lumos Orbitrap Mass Spectrometer, Novaseq, Hiseq
Data analysis	Flowjo v10, GraphPad Prism v9, Cellprofiler v4.2.4, HistoCat v1.761, MCD Viewer v1.0.560.6, R-package, DESeq2

For manuscripts utilizing custom algorithms or software that are central to the research but not yet described in published literature, software must be made available to editors and reviewers. We strongly encourage code deposition in a community repository (e.g. GitHub). See the Nature Research guidelines for submitting code & software for further information.

Data

Policy information about availability of data

All manuscripts must include a data availability statement. This statement should provide the following information, where applicable:

- Accession codes, unique identifiers, or web links for publicly available datasets
- A list of figures that have associated raw data
- A description of any restrictions on data availability

The data generated by this study are available in the manuscript and from the correspondig author on reasonable request. The accession number for the raw RNA-seq and ChIP-seq data reported in this paper is GEO: GSE150610 and EGAS00001005793. The alignment was performed using as a reference the human genome hg38 (genome assembly GRCh38.p13)
Raw Mass spectrometry data have been deposited to the ProteomeXchange Consortium (accession number: PXD024112)

Field-specific reporting

Please select the one below that is the best fit for your research. If you are not sure, read the appropriate sections before making your selection.

- [x] Life sciences
- [ ] Behavioural & social sciences
- [ ] Ecological, evolutionary & environmental sciences

For a reference copy of the document with all sections, see nature.com/documents/nr-reporting-summary-flat.pdf

Life sciences study design

All studies must disclose on these points even when the disclosure is negative.

Sample size	For in vitro studies, sample size was determined base on our previous experience. As a gold standard, minimum of n=3 was applied to all experiments performed with cell lines. Greater sample sizes were used for experiments with primary cells, largely based on the number of available samples. Number of biological replicates and sample size (n) is described in each figure legend. For animal studies sample size was chosen to comply with the 3R principles to minimize the number of mice used in the study.
Data exclusions	no exclusion criteria were applied
Replication	Sample size was chosen empirically and number of biological replicates and sample size (n) is described in each figure legend. In vivo 3 independent experiments were performed with multiple mice per group.
Randomization	For in vivo studies all mice were randomly allocated into each group without any pre-selection before the experiment. For in vitro studies no randomization was required as each dataset was internally controlled.
Blinding	For in vivo and in vitro studies investigators were not blinded. Automatic quantitative measures obtained by the relevant softwares used in this study did not require blinding. In addition, each experiment was performed using paired samples obtained from the same patient.

Reporting for specific materials, systems and methods

We require information from authors about some types of materials, experimental systems and methods used in many studies. Here, indicate whether each material, system or method listed is relevant to your study. If you are not sure if a list item applies to your research, read the appropriate section before selecting a response.

Materials & experimental systems

n/a	Involved in the study	
[ ]	[x]	Antibodies
[ ]	[x]	Eukaryotic cell lines
[x]	[ ]	Palaeontology and archaeology
[ ]	[x]	Animals and other organisms
[ ]	[x]	Human research participants
[x]	[ ]	Clinical data
[x]	[ ]	Dual use research of concern

Methods

n/a	Involved in the study	
[ ]	[x]	ChIP-seq
[ ]	[x]	Flow cytometry
[x]	[ ]	MRI-based neuroimaging

Antibodies

Antibodies used

PE/Cyanine7 anti-human CD274 (B7-H1, PD-L1) [Clone: 29E.2A3] Biolegend 329717 (1:100)
APC anti-human CD274 (B7-H1, PD-L1) [Clone: 29E.2A3] Biolegend 329708 (1:100)
PE/Cyanine7 anti-human HLA-DR Biolegend 307616 (1:300)
APC anti-human HLA-DR eBioscience 17-9956-41 (1:300)
PE anti-human CD8a Antibody [Clone: HIT8a] Biolegend 300908 (1:50)
APC/Cyanine7 anti-human CD4 Antibody [Clone: OKT4] Biolegend 317418 (1:50)
Anti-H3K27ac, clone: C15410196/pAb-196-050, Diagenode (1ug/IP)
Pacific Blue anti-human CD3 Antibody [Clone: HIT3a] Biolegend 300330 (1:50)
Ms CD45 BUV395 30-F11 50ug BD 564279 (1:100)
CD5-FITC BD 555352 (1:50)
CD19-APC BD 555415 (1:50)
Annexin-V APC Biolegend 640941
Ultra-LEAF™ Purified anti-human CD3 Antibody [Clone: OKT3] Biolegend 317325
Ultra-LEAF™ Purified anti-human CD28 Antibody [Clone: CD28.2] Biolegend 302933
Anti-CD19 (6OMP31)-142Nd Fluidigm 3142014D
Anti-Human CD4 (EPR6855)-156Gd Fluidigm 3156033D
Anti-Human CD8a (D8A8Y)-162Dy—25 µg Fluidigm 3162035D

Anti-Human PD-1 (EPR4877(2))-165Ho—25 µg Fluidigm 3165039D
Anti-Ki-67 (B56)-168Er—25ug Fluidigm 3168022D
Anti-Human PD-L1 (E1L3N)-150Nd—25 µg Fluidigm 3150031D
Cell-ID™ Intercalator-Ir—125um Fluidigm 201192A
Anti-Pan-Actin (D18C11)-175Lu—25 µg Fluidigm 3175032D
Anti-Histone 3 (D1H2)-176Yb —25 µg Fluidigm 3176023D
Human IFN-gamma R1 MAb (Clone 92101) R&D MAB6731-SP
B-Actin-HRP Cell Signalling 5125s (1:5000)
CIITA (7-1H), Monoclonal Antibody Insight Biotech SC-13556 (1:500)
Anti-Human IGM Stratech Scientific 109-067-043
Anti-hCD19-CD3 Invivogen BIMAB-HCD19CD3
Anti PDL1 (Durvalumab) Stratech Scientific A2013-SEL

Validation

All antibodies are commercially available and tested by manufacturers with detailed specificity described on their websites. Proper negative controls were used to guarantee specificity.
PE/Cyanine7 anti-human CD274 (B7-H1, PD-L1) [Clone: 29E.2A3] Biolegend https://www.biolegend.com/nl-be/products/pe-cyanine7-anti-human-cd274-b7-h1-pd-l1-antibody-8277

APC anti-human CD274 (B7-H1, PD-L1) [Clone: 29E.2A3] Biolegend 329708 https://www.biolegend.com/de-de/products/apc-anti-human-cd274-b7-h1-pd-l1-antibody-4376

PE/Cyanine7 anti-human HLA-DR Biolegend 307616 https://www.biolegend.com/fr-lu/products/pe-cyanine7-anti-human-hla-dr-antibody-2862

APC anti-human HLA-DR eBioscience 17-9956-41 https://www.thermofisher.com/antibody/product/HLA-DR-Antibody-clone-LN3-Monoclonal/17-9956-42

PE anti-human CD8a Antibody [Clone: HIT8a] Biolegend 300908 https://www.biolegend.com/fr-lu/products/pe-anti-human-cd8a-antibody-762

APC/Cyanine7 anti-human CD4 Antibody [Clone: OKT4] Biolegend 317418
https://www.biolegend.com/nl-nl/products/apc-cyanine7-anti-human-cd4-antibody-3658

Pacific Blue anti-human CD3 Antibody [Clone: HIT3a] Biolegend 300330
https://www.biolegend.com/fr-ch/products/pacific-blue-anti-human-cd3-antibody-6505

Ms CD45 BUV395 30-F11 50ug BD 564279
https://www.bdbiosciences.com/en-gb/products/reagents/flow-cytometry-reagents/research-reagents/single-color-antibodies-ruo/buv395-rat-anti-mouse-cd45.564279

CD5-FITC BD 555352
https://www.bdbiosciences.com/en-au/products/reagents/flow-cytometry-reagents/research-reagents/single-color-antibodies-ruo/fitc-mouse-anti-human-cd5.555352

CD19-APC BD 555415
https://www.citeab.com/antibodies/2412952-555415-bd-pharmingen-apc-mouse-anti-human-cd19

Annexin-V APC Biolegend 640941
Ultra-LEAF™ Purified anti-human CD3 Antibody [Clone: OKT3] Biolegend 317325
https://www.biolegend.com/nl-nl/products/ultra-leaf-purified-anti-human-cd3-antibody-7745

Ultra-LEAF™ Purified anti-human CD28 Antibody [Clone: CD28.2] Biolegend 302933
https://www.biolegend.com/it-it/products/ultra-leaf-purified-anti-human-cd28-antibody-7743

CIITA (7-1H), Monoclonal Antibody Insight Biotech SC-13556
https://datasheets.scbt.com/sc-13556.pdf

Anti-H3K27ac, Diagenode, clone C15410196; https://www.diagenode.com/en/p/h3k27ac-polyclonal-antibody-premium-50-mg-18-ml

Eukaryotic cell lines

Policy information about cell lines

Cell line source(s)

Mec1, Hg-3 (gift from IMS), Jeko1 (gift from DH), Lenti-x 293T (Takara 632180)

Authentication

No authentication test was performed

Mycoplasma contamination	Mycoplasma tests were performed every 3 months as per Institute's rules. All the tests were negative
Commonly misidentified lines (See ICLAC register)	Commonly misidentified lines were not used in this study

Animals and other organisms

Policy information about studies involving animals; ARRIVE guidelines recommended for reporting animal research

Laboratory animals	NOD.Cg-PrkdcscidIl2rgtm1Wjl/SzJ (NSG) were bred and maintained in our facilities (Cambridge University Biomedical Services). Only male 8-10 weeks old were used. All mice were maintained in a standard SPF facility (12 light/12 dark cycle, 19-23°C with 40-60% humidity). These animal studies have been regulated under the Animals (Scientific Procedures) Act 1986 Amendment Regulations 2012 following ethical review by the University of Cambridge Animal Welfare and Ethical Review Body (AWERB-PPL number P846C00DB).
Wild animals	Wild animals were not involved in this study
Field-collected samples	Field-collected samples were not involved in this study
Ethics oversight	These animal studies have been regulated under the Animals (Scientific Procedures) Act 1986 Amendment Regulations 2012 following ethical review by the University of Cambridge Animal Welfare and Ethical Review Body (AWERB-PPL number P846C00DB)

Note that full information on the approval of the study protocol must also be provided in the manuscript.

Human research participants

Policy information about studies involving human research participants

Population characteristics	After patients' informed consent and in accordance with the Helsinki Declaration, peripheral blood was obtained from adult patients with a diagnosis of CLL or MCL for experiment with live cells. Patients of any age and sex were included in these studies. For clinical data patients from the CLL-H2 study (Stilgenbauer et al., 2009) or the CLL Consortium (Consortium et al., 2010) were re-analyzed.
Recruitment	Patients were recruited by the Cambridge Biobank staff during routine outpatient visits. The authors were not directly involved in the selection of patients enrolled in the study. Recruitment for the clinical study was specified in Stilgenbauer et al., 2009 and Consortium et al., 2010
Ethics oversight	Studies were approved by the Cambridgeshire Research Ethics Committee (07/MRE05/44)

Note that full information on the approval of the study protocol must also be provided in the manuscript.

ChIP-seq

Data deposition

☒ Confirm that both raw and final processed data have been deposited in a public database such as GEO.

☒ Confirm that you have deposited or provided access to graph files (e.g. BED files) for the called peaks.

Data access links *May remain private before publication.*	https://www.ncbi.nlm.nih.gov/geo/query/acc.cgi?acc=GSE150584<br>Token mhkdcaqybvcfpgb
Files in database submission	file for each conditions analyzed is available.
Genome browser session (e.g. UCSC)	https://eu-central-1.protection.sophos.com?d=ucsc.edu&u=aHR0cHM6Ly9nZW5vbWUtZXVyby51Y3NjLmVkdS9zL1N0ZWxsYUNoYXJhbC9OQw==&i=NWZkYjRiODc3M2ZiN2EwZGZmZjA1YmJl&t=RmRsQ1ovTE1MRThnZGY4UGJzRHBacWF0QzBjMWdaVWt4Z2h1TlRXcjc0ST0=&h=00df0e2bab6b4746a30302f75304b343.

Methodology

Replicates	5 paired patients were analyzed
Sequencing depth	Sequencing was conducted in pair-ended manner
Antibodies	H3K27ac antibody, Diagenode: C15410196 /pAb-196-050
Peak calling parameters	Peaks of the H3K27ac data were called as described (http://dcc.blueprint-epigenome.eu/#/md/methods)
Data quality	MACS2 was used for the peak calling of the samples
Software	deseq2 was used for the normalization of the peak counts

Flow Cytometry

Plots

Confirm that:

- [x] The axis labels state the marker and fluorochrome used (e.g. CD4-FITC).
- [x] The axis scales are clearly visible. Include numbers along axes only for bottom left plot of group (a 'group' is an analysis of identical markers).
- [x] All plots are contour plots with outliers or pseudocolor plots.
- [x] A numerical value for number of cells or percentage (with statistics) is provided.

Methodology

Sample preparation	Cells were stained with fluorophore-labelled antibodies in 2% BSA in PBS according to the manufacturer’s instructions. For apoptosis analysis, conjugated Annexin-V and DAPI were used for the detection of apoptotic cells according to the manufacturer’s instructions. Cell cycle analysis was performed using the Click-iT™ EdU Alexa Fluor™ 647 Flow Cytometry Assay Kit (ThermoFisher Scientific) according to manufacturer’s instructions
Instrument	FACS acquisition was performed using BD Fortessa x-20. All cell sorting was performed using BD Influx cell sorter
Software	Data acquisition was done using DIVA software and data analysis using FlowJo
Cell population abundance	FACS plots show the same population abundance.
Gating strategy	Dead cells, debris and doublets were excluded by SSC FCS and DAPI staining profile.

- [x] Tick this box to confirm that a figure exemplifying the gating strategy is provided in the Supplementary Information.
